# Microscopic mammalian retinal pigment epithelium lesions induce widespread proliferation with differences in magnitude between center and periphery

**Published:** 2010-03-31

**Authors:** Peter Lundh von Leithner, Coziana Ciurtin, Glen Jeffery

**Affiliations:** 1 Institute of Ophthalmology, University College London, UK; 2 Faculty of General Medicine, Carol Davila University of Medicine and Pharmacy, Rumania

## Abstract

**Purpose:**

The vertebrate retina develops from the center to the periphery. In amphibians and fish the retinal margin continues to proliferate throughout life, resulting in retinal expansion. This does not happen in mammals. However, some mammalian peripheral retinal pigment epithelial (RPE) cells continue to divide, perhaps as a vestige of this mechanism. The RPE cells are adjacent to the ciliary margin, a known stem cell source. Here we test the hypothesis that peripheral RPE is fundamentally different from central RPE by challenging different regions with microscopic laser burns and charting differential responses in terms of levels of proliferation and the regions over which this proliferation occurs.

**Methods:**

Microscopic RPE lesions were undertaken in rats at different eccentricities and the tissue stained for proliferative markers Ki67 and bromodeoxyuridine (BrdU) and the remodeling metalloproteinase marker 2 (MMP2).

**Results:**

All lesions produced local RPE proliferation and tissue remodeling. Significantly more mitosis resulted from peripheral than central lesions. Unexpectedly, single lesions also resulted in RPE cells proliferating across the entire retina. Their number did not increase linearly with lesion number, indicating that they may be a specific population. All lesions repaired and formed apparently normal relations with the neural retina. Repaired RPE was albino.

**Conclusions:**

These results highlight regional RPE differences, revealing an enhanced peripheral repair capacity. Further, all lesions have a marked impact on both local and distant RPE cells, demonstrating a pan retinal signaling mechanism triggering proliferation across the tissue plane. The RPE cells may represent a distinct population as their number did not increase with multiple lesions. The fact that repairing cells were hypopigmented is of interest because reduced pigment is associated with enhanced proliferative capacities in the developing neural retina.

## Introduction

The vertebrate retina develops with a center to periphery gradient, such that the first cells to be generated are at the caudal pole and the last are at the far periphery next to the ciliary margin. In fish and amphibians cell production is maintained at the retinal rim throughout life, resulting in retinal expansion [1]. In mammals retinal proliferation ends during development, although the ciliary margin remains a source of stem cells, perhaps reflecting mechanisms found in lower vertebrates [2]. Additionally, it has been demonstrated in rat and man that the retinal pigment epithelium (RPE) can be subdivided into at least two regions [3]: a peripheral region adjacent to the ciliary margin where there is persistent cell production throughout life and a senescent central area. Some peripheral RPE appears to divide repeatedly, and there is evidence that some cells migrate centrally [3,4]. Taken together, these data are consistent with the notion that the peripheral RPE–ciliary margin region is an area that retains a developmental capacity into adulthood in mammals.

The RPE is capable of repair. Lesions of the RPE cells can result in local proliferation [5–7]. In light of the demonstration that the RPE can be divided into proliferating and senescent regions [3], we ask whether the cellular response to injury is fundamentally different between central and peripheral retina following microscopic local laser burns and how far this response extends through the tissue plane. We reveal enhanced responses to injury in peripheral RPE. We also demonstrate that a single microscopic lesion can induce RPE proliferation across the entire RPE surface.

## Methods

We made small low-energy laser lesions of the RPE at defined locations in rats. The tissue was then examined with a range of techniques both in vivo and in vitro. Three time periods were used. A preliminary assessment was made to define the nature of the damage induced (6 h to 7 days). However, the majority of the analysis was undertaken 3 days post lesion, as preliminary experiments indicated that this period coincided with peaks in RPE cell production and provided the clearest window on repair mechanisms. Exceptions to this were bromodeoxyuridine (BrdU) pulse-chase experiments undertaken 1–9 days post lesion to trace proliferating RPE cells. Finally, the RPE was examined at 12 weeks post lesion to assess the extent of tissue repair. The number of animals used in different experimental groups is given in Table 1.

**Table 1:** Experimental animal groups.

**Experiment**	**Lesions**	**Animals**	**Processing**
Wound development	1 to 4 per eye	18	In vivo imaging, MMP-2, Caspese-3
Cell proliferation	1 to 4 per eye	34	K_i_-67, BrdU, MMP-2, Otx2
Wound repair	2 per eye	8	ZO-1, Otx2

Wherever possible individual animals were used in multiple experiments and eyes harvested bilaterally. Abbreviations: BrdU represents bromodeoxyuridine, MMP2 represents metalloproteinase marker 2, Otx2 represents orthodenticle homeobox 2, ZO-1 represents zona occludens 1.

### Animals

Two-month-old (n=50) Dark Agouti (DA) rats (175–200 g bodyweight [BW]) were used. These are an inbred stain supplied by Harlan (Bicester, UK). Animals were anesthetized by intraperitoneal (IP) injection of Ketaset (37.5%; Fort Dodge Animal Health Ltd, Southampton, UK), Dormitor (25%; Pfizer Animal Health, Kent, UK), and sterile water (Norbrook Laboratories Ltd, Carlisle, UK) at 0.2 ml/100 g. Before laser lesioning or imaging, pupils were dilated with phenylephrine hydrochloride 2.5% and tropicamide 1.0% (Chauvin Pharmaceuticals Ltd, Kingston-Upon-Thames, UK). If animals recovered, anesthesia was reversed by IP injection of Antisedan (20%), (Orion Pharma, Espoo, Finland) and sterile water at 0.01 ml/150 g. All animals under anesthesia were kept warm on a 37 °C heating pad. The number of animals used in each procedure is given in Table 1. All animal procedures were approved by the British Home Office (Animals Scientific Procedures Act 1986) and were in compliance with University College London local ethical committee regulations.

### Laser generated lesions

Retinas of all rats were exposed to laser photocoagulation (PC) to cause focal RPE lesions. The burns were applied either bilaterally or unilaterally using a diode-pumped laser (Novus Omni, Coherent Inc., Santa Clara, CA) attached to a slit-lamp funduscope and a hand-held planoconcave contact lens applied to the cornea together with Viscotears Liquid Gel (BR Lewis Pharmaceutical Ltd, London, UK) to neutralize ocular power.

**Figure 1:**
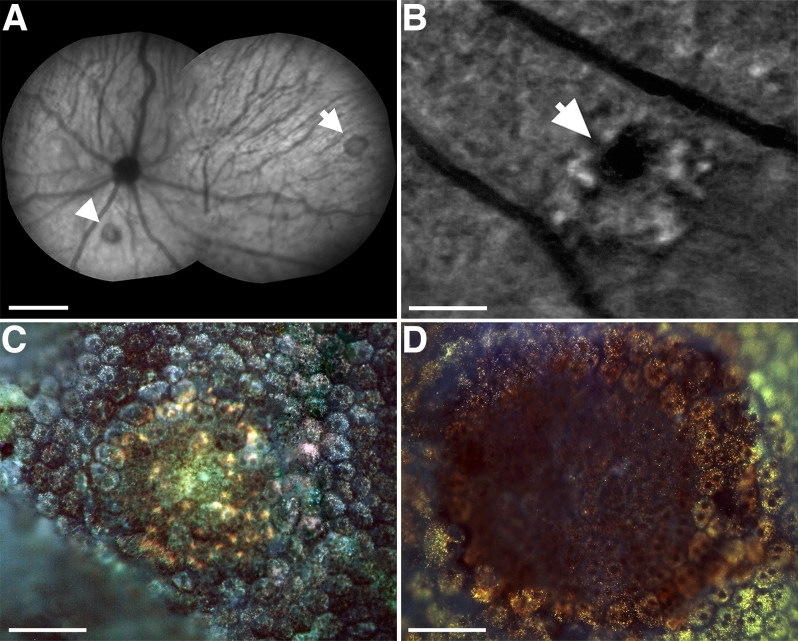
In vivo and histological fluorescence images exhibiting the impact of photocoagulation on retinal pigment epithelium. **A**: In vivo near-infrared reflectance image made immediately after photocoagulation (PC) showing placement of central (arrowhead) and peripheral (arrow) lesions in the retina. Scale bar represents 1 mm. **B**: In vivo autofluorescence image of a lesion made 7 days after PC shows absence of fluorescence at the center of the lesion. The lesion is surrounded by hyperfluorescent point sources. Scale bar represents 200 µm. **C**: Autofluorescence image of a lesion in retinal pigment epithelium (RPE), which was excised and flatmounted 6 h after PC. The RPE tissue is continuous across the lesion site, consistent with the notion that PC did not result in immediate tissue destruction. **D**: Autofluorescence image 72 h after PC, showing a clear lesion site and the absence of tissue. Scale bars in **C** and **D** represent 50 µm.

Between one and four lesions (633 nm, 80 mW, 0.2 s, and 100 µm diameter) were distributed in central and peripheral retina. Central lesions were produced at <0.5 mm radius from the optic disk, while peripheral lesions were placed at a minimum radius of >2.0 mm from the optic disk. These low-level lesions immediately produced an opaque retinal spot (Figure 1A) that disappeared within 1 h. The pathological end point of the laser injury was to damage RPE cells in a defined region while causing minimal disruption to the underlying Bruch’s membrane and the overlaying neural retina. The region of damage was examined after lesioning, using a confocal laser ophthalmoscope (cSLO). If a lesion accidentally produced a gaseous bubble, indicating a rupture of Bruch’s membrane, or hemorrhage, the retinas were discarded from the experiment.

### In vivo imaging

A modified cSLO (HRA2 Heidelberg-Engineering, Heidelberg, Germany) was used to image lesioned retinas in vivo. The cSLO was modified to work with the shorter optical distances found in rodents compared with man by the application of front-end lenses. The axial and lateral resolution of cSLO images were 15.7 µm and 2.2 µm, respectively. The power of the Argon-ion laser excitation was 250 mW (measured at the rat pupil). A second near-infrared laser line was also used to map lesions (power 70 mW at the pupil). The cSLO frame rate was 8.9 Hz, and the field-of-view was 55°. To avoid cataract formation and other ocular artifacts, the rodent corneas were kept moist during imaging by using 2% hydroxypropylmethylcellulose (Torbay P.M.U., Devon, UK).

### Histology and imunohistochemistry

At termination of experimental procedures and where histological post processing was required, rats were terminally anesthetized by IP injection using a mixture of Ketaset (50.0%), Domitor (33%), and sterile water (0.4 ml/100 g) and perfused intracardially with 0.1 M PBS, (catalog number E404–100T-R, Amresco, Solon, OH), followed by 4% paraformaldehyde in 0.1 M PBS at 6 h, 1, 3, 5, 7, 9, and 90 days post lesioning. Eyes were enucleated and placed in fixative for 2 h at 4 °C. The anterior chamber, lens, and neural retina were removed to produce eyecups where the RPE was exposed. To obtain wholemounts, four equidistant cuts were made in the eyecups. Autofluorescence micrographs were produced using an excitation wavelength of >498 nm of the RPE sheet before immunohistochemistry. A list of the primary antibodies used and their dilutions is given in Table 2.

**Table 2:** Antibodies used for immunohistochemistry and their working dilutions.

**Antibody**	**Manufacturer**	**Catalog number**	**Dilution**
Rabbit PaB to K_i_-67	Vectorlabs	VP-K451	1:2,000
Rabbit PaB to Otx2	Chemicon/Millipore	ab9566	1:1,000
Rabbit PaB to ZO-1	ZYMED Laboratories, Invitrogen	40–2200	1:100
Mouse MaB to MMP-2	Abcam	ab7032	1:200
Rabbit PaB Caspese-3	Abcam	Ab2302	1:100
Mouse MaB to BrdU	[11]	-	1:5

Anti-BrdU was produced using a hybridoma cell line [11]. Abbreviations: BrdU represents bromodeoxyuridine, MMP2 represents metalloproteinase marker 2, Otx2 represents orthodenticle homeobox 2, ZO-1 represents zona occludens 1.

The eyecups were placed in a multichamber slide well and processed for immunoﬂuorescence, using multiple antibodies (Table 2). The tissues were first blocked with 5% normal donkey serum (NDS; Jackson ImmunoResearch, West Grove, PA) in PBS (as above) for 2 h at room temperature, followed by overnight application of primary antibodies diluted in PBS with 1% NDS at room temperature. The following primary antibodies were used for immunohistochemical characterization of cell death, differentiation, and proliferation: MMP2 [8], Ki67, caspase-3, BrdU [3], orthodenticle homeobox 2 (Otx2) [3], and zona occludens 1 (ZO-1) for junctional marking [9]. After washing, secondary antibodies were applied in PBS plus 2% NDS. The secondary antibodies used (1:2,000, preadsorbed to various species, including rat, mouse, and human) were Alexafluor 488 and 593 donkey antigoat, antimouse, and anitrabbit Immunoglobulin G (IgG; SantaCruz Biotechnology, Inc., Santa Cruz, CA). After washing, cell nuclei were counterstained with 4’6-diamindino-2-phenylindole dihydrochloride (Sigma-Aldrich, Pool Dorset, UK), washed in PBS and Tris buffer (0.05 M, pH 7.4), and coverslipped with Vectashield (Vector Labs, Peterborough, UK). In some cases flatmounted tissue was removed from slides after imaging. Lesions were identified and excised from wholemounts using an operating stereomicroscope. These segments were cryoprotected in 30% sucrose, then snap frozen, cryostat sectioned at 10-µm thickness, and imaged again in transverse section.

### Quantification of cell proliferation using bromodeoxyuridine

BrdU (5-bromo-2-deoxyuridine; Sigma Aldrich) was used to detect proliferating RPE cells and to confirm that complete cell division was taking place, not just cell-cycle entry or cell-cycle retention [10]. Two experiments were performed investigating potential increases in RPE proliferation after lesioning. First, proliferating cells were quantified as a function of radial distance from the optic disk, using a combination of central and peripheral lesions. DA rats (n=8) were given IP injection of BrdU (50 μg/g BW) in PBS. Second, to ensure that all dividing cells were labeled with BrdU, animals were given six successive IP injections at 7-h intervals over a 48-h period 3 days following the lesion. Animals were killed via CO_2_ exposure and perfused 1 h after the last BrdU injection, and retinas stained immunohistochemically with a monoclonal antibody to BrdU [11]. Third, pulse-chase BrdU experiments to examine cell proliferation as a function of time after RPE injuries were performed. The RPE of DA rats (n=10) was lesioned and immediately followed with a single dose of BrdU (200 µg/g BW). The first group of rats was killed 1 day after the BrdU injection. Groups were then sacrificed at 2-day intervals up to 9 days post lesion to provide an index of RPE cell proliferation.

### Statistical analysis

Differences in the populations of proliferating RPE cells within and at radial intervals from lesions in the center and periphery of the pigment epithelium were statistically quantified using one-way ANOVA (ANOVA; *F, P*) followed by post hoc comparison using the Student *t* test for unpaired data (*t, P*). One-way ANOVA (*F, P*) was used to identify significant time points of proliferation in the pulse-chase experiments, which were thereafter verified post hoc using the Student *t* test for unpaired data (*t, P*). Correlation between different immunohistochemistry antibody expression patterns was quantified using Pearson’s correlation coefficient (*r*). An α-level equal to 0.05 was used as significance criterion in all tests. All data analyses were performed using Excel 2008 and PASW v.18 (SPSS Inc., Chicago, IL).

## Results

### Lesions

Laser burns could be identified in vivo using cSLO imaging immediately after they were made. Laser burns were approximately 100 µm in diameter independent of location and resulted in a region of increased RPE autoflorescence that was apparent within a 6 h after the lesion in vitro. At this stage RPE cells could be identified in vitro within the lesioned region and were continuous across this area, with no obvious sign of cell loss. Hence, the energy applied did not result in immediate tissue destruction. However, when the tissue was examined 72 h later in vitro, there was a clear hole in the RPE centered on the lesion site. This pattern was consistent between lesions placed at different retinal locations (Figure 1).

**Figure 2:**
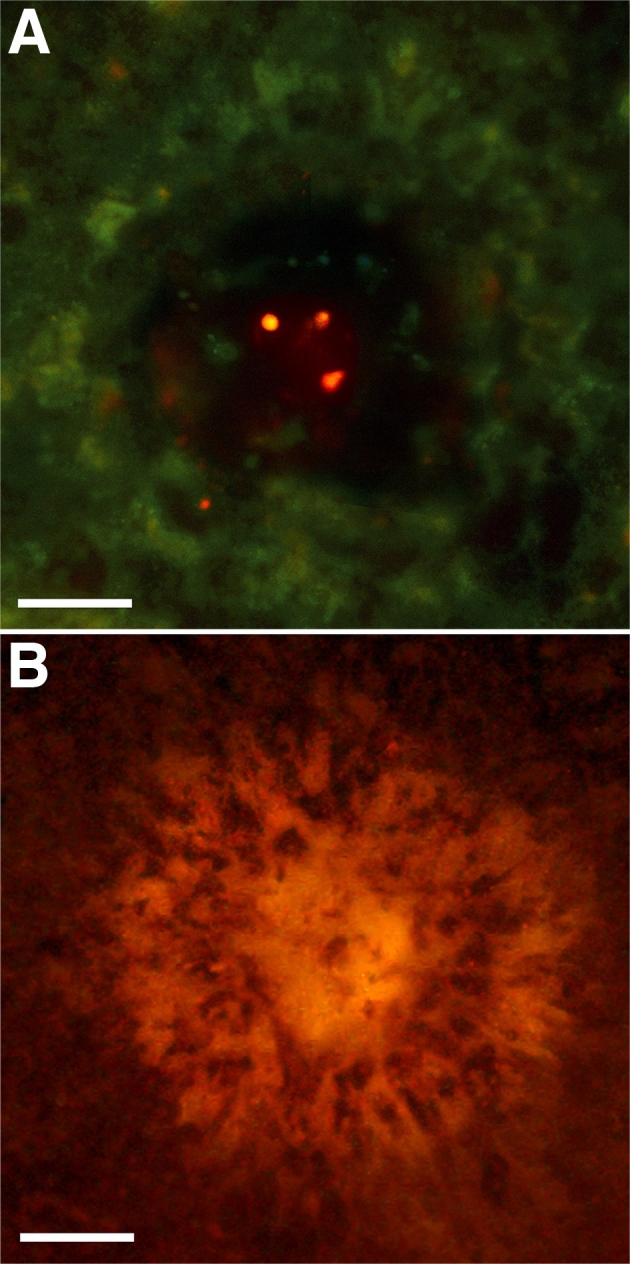
Immunostaining of retinal pigment epithelium lesion site 72 h after injury. **A**: Immunoreactivity for activated caspase-3 in lesioned retinal pigment epithelium cells demonstrated that at this stage cell death was still a feature of the tissue. **B**: Expression of metalloproteinase marker 2 was consistent with the process of extracellular degradation and tissue remodeling. Scale bar represents 50 µm.

Examination 72 h post lesion was combined with staining for caspase-3 activity, a marker of cell death. This demonstrated the presence of caspase-3-positive cells within the lesion but not beyond. There were no differences in such patterns of labeling between lesions in central or peripheral areas (Figure 2A). At this time point, the tissue was also stained with MMP2, which marks the breakdown/remodeling of the extracellular matrix. Positive staining for this was also found in and around the lesion sites (Figure 2B). The positive staining for MMP2 is also consistent with the process of tissue remodeling within the damaged region and is the probable reason why positive staining for this extended beyond the limits of the immediate lesion site. MMP2 staining could commonly be found extending into areas three times the diameter of the actual lesion. When central and peripheral lesions were compared, the area of MMP2 staining appeared larger in peripheral regions compared to central regions. However, the nature of the staining pattern with its relatively soft edges mitigated against quantifying such differences.

### Retinal pigment epithelium proliferation

Lesions were targeted at peripheral and central locations and assessed 72 h later for the upregulation of the cell-cycle marker Ki67, and subsequently with BrdU, to reveal patterns of cell production. This period had been identified in preliminary experiments as the most suitable for assessing patterns of RPE proliferation because they peaked around this time window.

**Figure 3:**
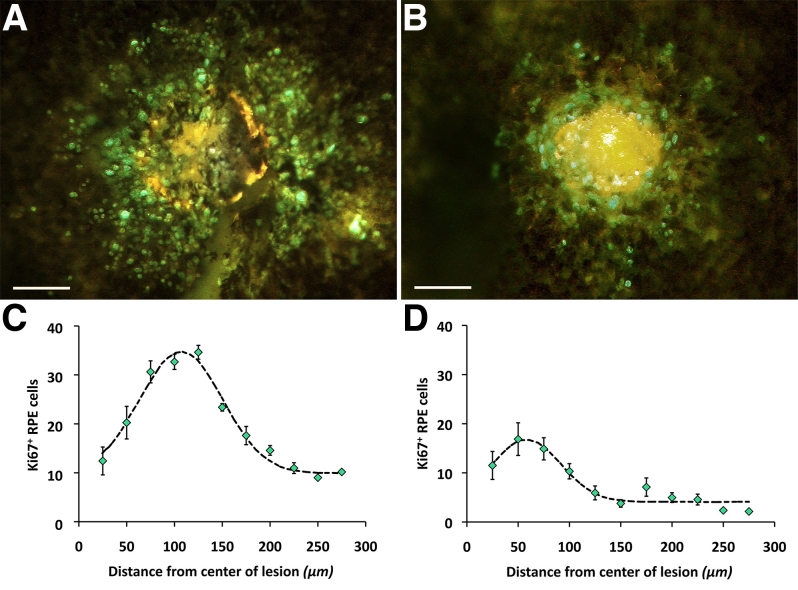
Levels of proliferation determined by Ki67 positive label in central and peripheral regions. But there were significantly higher levels of proliferation in retinal periphery (**A** and **C**) compared to central retinas (**B** and **D**). The graphs show the mean levels of Ki67 positive cells in progressive annular regions around lesions. Differences were statistically significant. Scale bars represent 100 µm.

Irrespective of location, all lesions were associated with an upregulation of Ki67-positive cells in the RPE sheet. Significantly more Ki67-positive cells were found around peripheral lesions than those located centrally (*t*=7.43, p<0.001). Figure 3 shows the number of Ki67-positive cells in progressive intervals around central and peripheral lesions of the same size. Further, Ki67 labeling was mainly confined to the edge of the lesion in those located centrally, peaking at approximately 50 µm from the lesion center. In peripheral lesions the spread of Ki67-labeled cells extended over a wider region, peaking approximately 100 µm from the lesion center. An additional feature of such labeling was that Ki67-positive RPE cells were also present beyond the immediate lesion area, being present across the entire retina. Hence, unexpectedly, lesions increased Ki67 labeling pan retinally.

**Figure 4:**
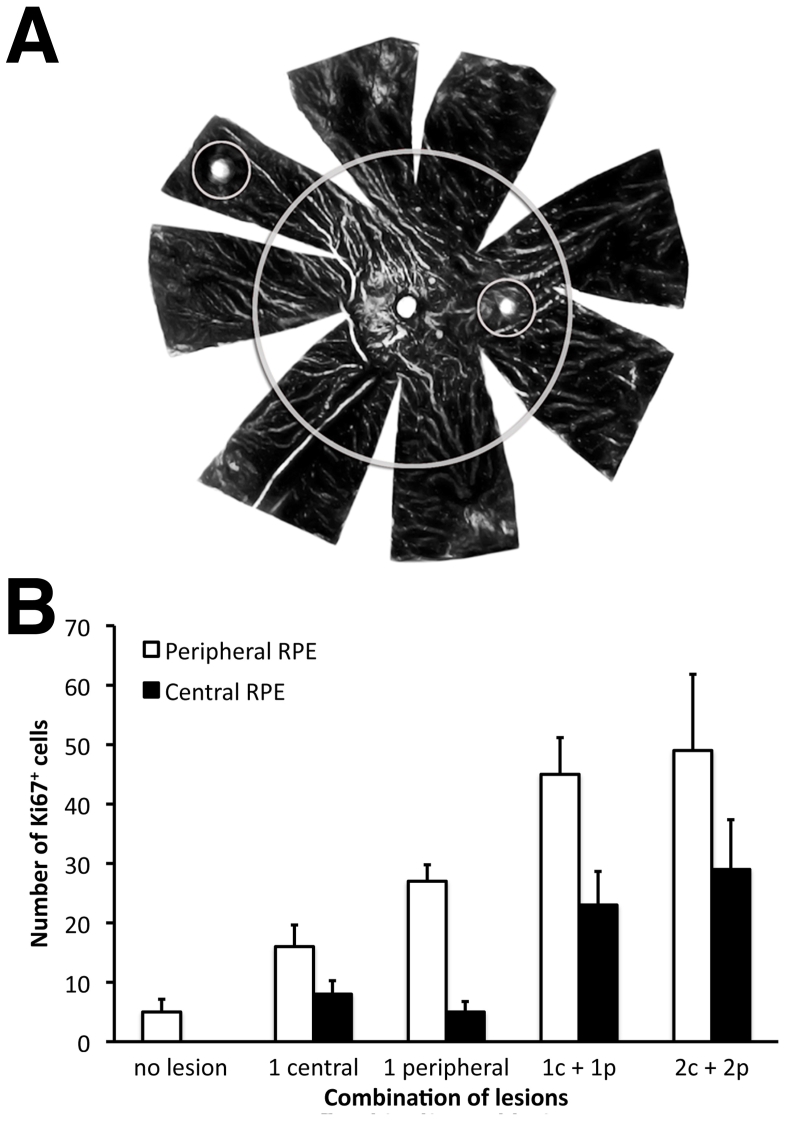
The number of retinal pigment epithelium (RPE) cells that had entered the cell cycle 72 h after lesioning were not directly associated with the lesion site. **A**: Schematic representation of lesion configurations and demarcation of center from periphery on an RPE tissue sheet. **B**: Total cell numbers were counted in central and peripheral areas, both in terms of those directly associated with a lesion and those distant from lesions. Different combinations of lesions were applied. First, no lesion (control), which represents Ki67 numbers in normal RPE. Second, cell numbers found after one central lesion. Third, cell number after one peripheral lesion. Finally numbers following after one central (c) and one peripheral (p) lesion, and finally numbers following two central and two peripheral lesions. Irrespective of the lesion configuration, elevation in Ki67 positive cell number was always greater was always greater in the periphery, even when only one central lesion was made. While Ki67 numbers increased with lesion number, the number of Ki67 expressing cells found following two lesions in each area was not greater than when one lesion was made in each area. The progressive elevation in cell numbers was statistically significant between no lesions and 1c+1p lesion (see the text for statistical significance).

To reveal the relative influence of central and peripheral lesions on Ki67 upregulation in local and distant labeling patterns, single and multiple lesions were placed in these different locations in four groups of animals (Figure 4). Each group contained four eyes, and labeled RPE cells were counted across the entire center and the entire periphery but not in the immediate vicinity of the lesion, determined as a radial distance of 300 µm from the lesion center. There was a significant increase in RPE cell proliferation in both peripheral and central retinas with increasing number of lesions (ANOVA; periphery *F*=8.56, p<0.001; center *F*=7.63, p<0.001; Figure 4). In the first group, a single central lesion was made. This resulted in Ki67 upregulation in both central and peripheral areas, more than doubling the number normally found in the periphery and lifting levels in the center to approximately the number found in the normal nonlesioned peripheral retina. However, the differences between center and periphery were not statistically significant (*t*=2.99, p<0.06). The same size lesion placed in the periphery resulted in a very substantial increase in the number of Ki67-positive cells in peripheral regions but also upregulated numbers in the center, although to a level less than that found following a single central lesion. Differences between center and periphery following this pattern of lesioning were statistically significant (*t*=19.46 p<0.0003).

Combining a central lesion with one in the periphery again increased the total number of cells in these regions, although levels in the periphery were always significantly enhanced compared with the center (*t*=4.67, p<0.02). When two lesions were made in the center and two in the periphery, the number of Ki67 cells found did not increase significantly beyond that with one lesion in each area (center *t*=1.31, p<0.28; periphery *t*=1.00, p<0.39). However, in both cases when the center was compared with the periphery, more cells were always present in the periphery; in the cases of 2+2 lesions, the difference was not significant (1+1 lesion *t*=4.67, p<0.02; 2+2 lesion *t*=2.24, p<0.11). Hence, the number of Ki67-positive cells found beyond lesion sites appears to have an upper limit. This is supported by experiments where more than four lesions were made (data not shown). Given this result, it is possible that these proliferating cells form a distinct RPE cell population.

**Figure 5:**
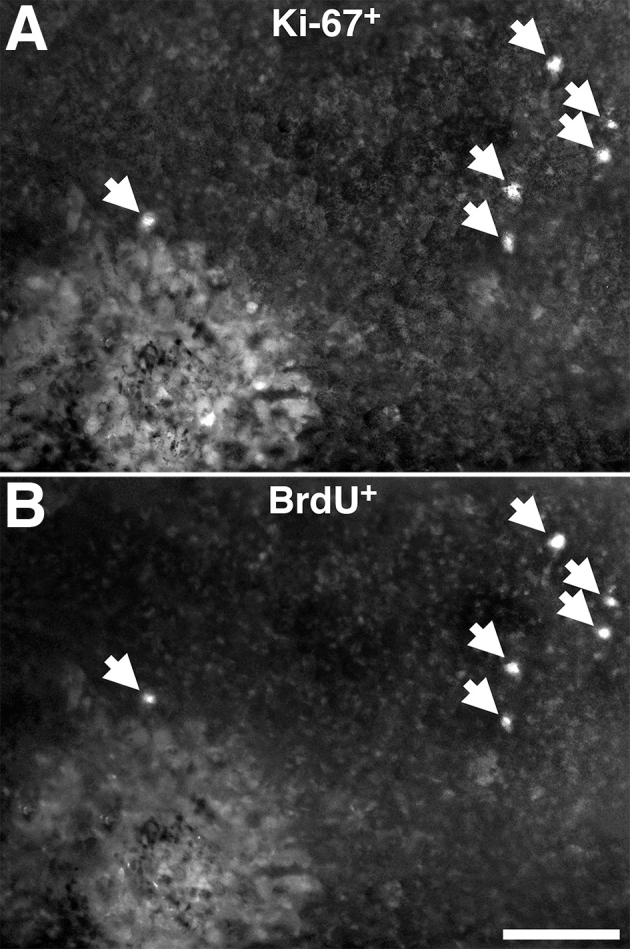
Double labeling combining Bromodeoxyuridine (BrdU) and Ki67 protocols 72 h following photocoagulation (PC) lesioning of the RPE with one central and one peripheral lesion. **A** shows labeling for Ki67 and **B** for BrdU in the same tissue. Arrows indicate cell labeled with both markers. The data revealed extensive co-localization of both labels, confirming that many of the cells that were positive for Ki67, and as such in the cell cycle, were also positive for BrdU, confirming that full cell division had taken place. More cells positive for Ki67 and BrdU were always found in the periphery than in the center, reflecting patterns found in Figure 3 and Figure 4 (data not shown). The scale bars represent 100 µm.

Ki67 is a cell-cycle marker [12]. To confirm that RPE cells were actually proliferating rather than simply entering the cell cycle, retinas with one central (n=4) or one peripheral (n=4) lesion were double labeled with BrdU and Ki67 or BrdU and Otx2. In and around the lesion, numerous Ki67-positive cells could be found, but few were positive for BrdU within the lesion at 72 h post lesion. However, in the region beyond the lesion, cells were positive for both markers, with a high correlation of double-labeled cells (correlation coefficient r=0.96) (Figure 5). Hence, cells were entering the cell cycle and completing cell division. These cells were also positive for Otx2, confirming that they were RPE cells (data not shown).

**Figure 6:**
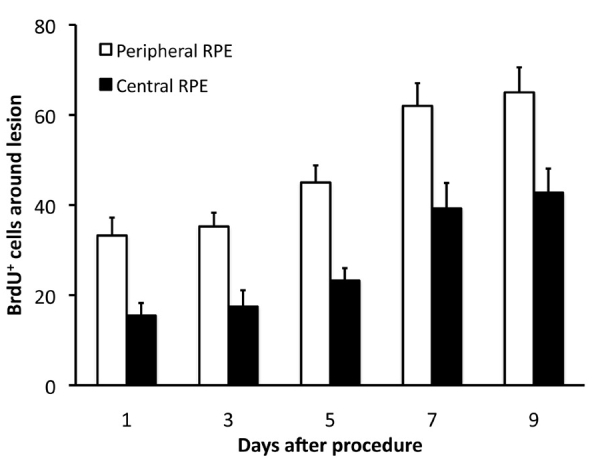
Bromodeoxyuridine (BrdU) pulse chase experiment in animals having one central and one peripheral photocoagulation (PC) lesion followed by a single pulse of BrdU. The number of labeled cells increased over days 1–7, then leveled off between days 7–9. Levels of BrdU labeling in each case were greater in the periphery than in the center. The increasing number of cells found over time was statistically significant (see test for). These data reveal that cell numbers double approximately between day 1 and day 7; hence the cell-cycle rate is likely to be roughly this long.

Pulse-chase experiments were undertaken to chart the progress of cell production after a single lesion in the center and one in the periphery. A single injection of BrdU was administered immediately after lesioning. Here, animals were killed at progressive 2-day stages from day 1 to day 9 (n=4 at each time point). This configuration of lesions was chosen to maximize the number of cells that could be chased with minimal damage. Lesions were always placed as in Figure 4A. These data provide a progressive temporal picture of patterns of cell production rather than simply which cells enter the cell cycle (Figure 6). These data include all central and peripheral cells. Cells continued to divide, increasing their number significantly from day 1 through to the end of day 9 in both subregions (ANOVA; center *F*=8.93, p<0.001; periphery *F*=11.42, p<0.0002). At each stage there were significantly more labeled cells in the periphery compared with the center (day 1 *t*=6.90, p<0.01; day 3 *t*=7.57, p<0.005; day 5 *t*=9.77, p<0.002; day 7 *t*=5.44, p<0.01). Beyond day 9, label intensity within cells declined markedly, presumably due to dilution following multiple divisions. This was not the reason for the leveling off in label at day 7, as cells here were as bright as previously. These data also show that cell numbers double around 1 week post injection, implying a cell-cycle rate similar to that found in uninjured RPE [3,10]. BrdU-labeled cells were common in the center of the lesion at 1–2 days post injection but did not increase after this. This was why they were not identified here when BrdU was injected at 3 days post lesion (above and Figure 5).

### Retinal pigment epithelium repair

**Figure 7:**
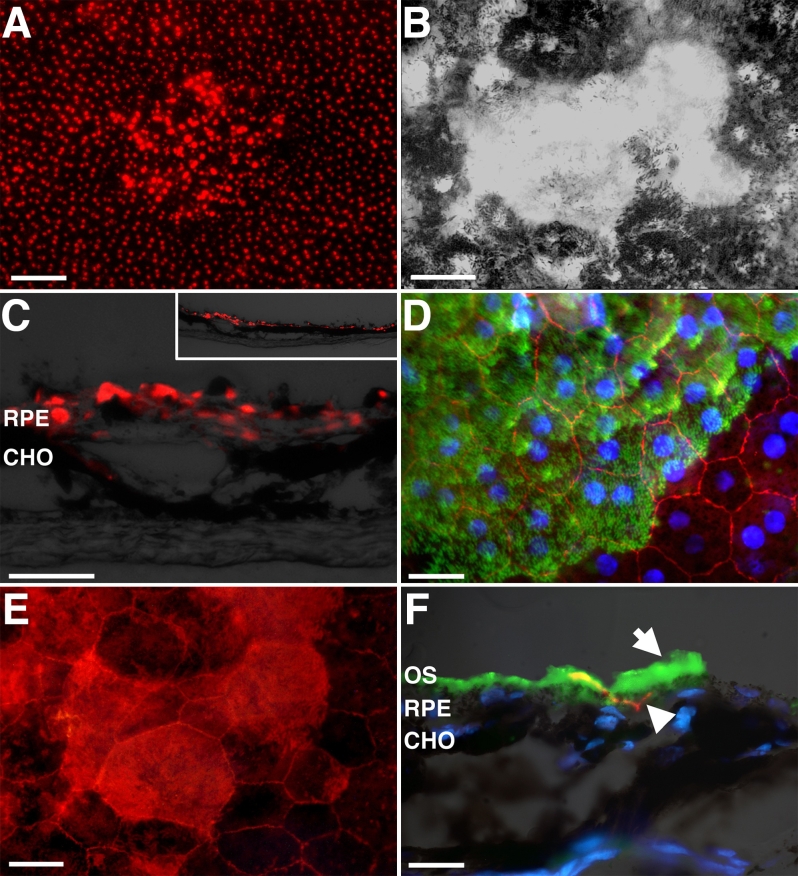
Three months after lesioning both central and peripheral injuries caused by photocoagulation (PC) were repaired with new retinal pigmented epithelium (RPE) cells that expressed RPE specific markers. **A**: Otx2 expression marking RPE cells was continuous across the lesion in whole mounted preparations. The scale bar here represents 100 µm. **B**: Reveals that repaired regions were hypopigmented with many RPE cells either lacking pigment completely or containing reduced pigment levels. **C**: Otx2 expression in RPE cells was also continuous across lesions when they were viewed in section. This image shows the location of these cells in relation to the choroidal capillaries (CHO). The scale bar is 25 µm and the image also contains a low power figure of the region. **D**: The RPE tight junction protein ZO-1 (red) was also present across the lesion site. The nuclei here are shown in blue. Unexpectedly, photoreceptor outer segments (green) remained adhered to the repaired tissue. Their location here demarcates the lesion site. The scale bar here represents 50 µm. **E**: Shows a high power image of a lesion site revealing Otx2 expression and the brighter cells inside due to the absence of melanin. The scale bar here is 20 µm. **F**: A transverse section through the repaired region of the RPE. The green outer segments (OS) are indicated by the upper arrow. The lower arrow head reveals the ZO-1 (red). The location of the CHO is also indicated under the other tissues. Nuclei are blue. The scale bar is 20 µm.

Lesioned sites were examined at 3 months to characterize the nature of the repair. In both central and peripheral retina the small lesions had fully repaired. Cells positive for the RPE cell marker Otx2 were present across the lesions site when tissue was viewed in section or en face (Figure 7A,B). These Otx2-positive cells were continuous with those around the lesion, although they appeared less regular in mosaic order and were larger. Cells within the lesion were also consistently brighter when labeled with Otx2 than those in the surrounding tissue (Figure 7A,C).

A distinct feature of the repaired region was that many of its constituent cells were either hypopigmented or albino. There was considerable variability in the extent of this, with some cells still containing some pigment while others were pigment free (Figure 7E). The reason why cells labeled with Otx2 were brighter in lesions than in surrounding tissue was probably because melanin granules, which have a light-filtering influence, were absent. It is probable that the RPE cells that proliferated divided their melanin content between progeny, and this resulted in the albino-like, hypopigmented cells identified within the lesion site.

RPE cells within the lesion site established relatively normal patterns of connectivity, as revealed by ZO-1 labeling at 3 months. It was also clear that with the low-powered lesions used here that the neural retina over the lesions was relatively normal histologically, having its full complement of cellular layers and outer segments (data not shown). The only distinguishing feature of the lesioned region was that when the retina was dissected from the RPE following fixation, the repaired region consistently retained photoreceptor outer segments, which in these preparations had a green autofluorescent appearance. This was apparent in both wholemounted preparations and in sectioned tissue (Figures 7E,F). This implies that although the tissue had repaired, it lacked the normal relationship with the outer neural retina, which is probably because the apical processes of RPE cells that extend from the RPE and are responsible for removing photoreceptor outer segments are more extensive in lesioned area than in repaired regions, although the reason for this is unclear.

## Discussion

Many investigators have examined lesions of the RPE and the repair of this tissue [5,13]. However, we have recently identified key differences in the proliferative capacity of peripheral compared to central RPE and exploited this information to divide an analysis of RPE repair into these subregions [3]. The key finding demonstrated here is that peripheral RPE displays an enhanced response to local injury compared with the response in central regions. We also show unexpectedly that small lesions induce RPE cell division globally. RPE cell division continues for approximately 9 days, with a cell-cycle rate in many cells of around 5–7 days, and the repaired tissue has markedly reduced pigmentation.

The lesions generated in our study were small both in terms of their area and the energy delivered. They did not result in immediate tissue destruction; rather damage became apparent a few days after the laser burn. Additional evidence that the laser damage was minimal comes from the finding that there was at no point any indication of choroidal neovascularization, which is the common outcome of laser lesions disrupting Bruch’s membrane [14]. Also, outer segments were present over repaired regions, indicating that damage to the neural retina was minimal. Outer segments would not have regenerated had the laser burn included photoreceptor nuclei in the outer nuclear layer; this would have led to photoreceptor destruction. These features provide strong evidence that the lesions applied here disrupted RPE cells specifically and selectively, leaving adjacent tissue intact.

We used Ki67 as a key marker of cell-cycle entry in the RPE [3]. However, cells can express this marker during apoptosis [15,16], hence we also used BrdU, a marker of proliferation. In spite of the association between cell death and Ki67, it is unlikely that the positive Ki67 label was due to cell death. No caspase-3 label was found outside the center of a lesion. The majority of Ki67 label was around the edge of the lesions and in distant regions. Hence, the distribution of the two labels was separate, and consequently cells labeled with Ki67 were unlikely to be dying. Further, although BrdU labels different populations of cells compared with Ki67, the two markers showed a significant degree of co-localization.

Why is there a differential capacity to repair between the center and the periphery? It has been demonstrated in the same rat strain used here [3] that the mature peripheral RPE contains a population of cells retained in the cell cycle. These number approximately 20 at any time point and have a cell-cycle rate of about 5–7 days. These cells are not found centrally. Further, it is known that different genes are expressed between central and peripheral RPE and that some of those expressed only in the periphery relate to cell-cycle regulation [17]. This provides supportive genetic evidence for our results. However, we do not know how these patterns of gene expression change following RPE site-specific lesions.

Del Priore and coworkers proposed that central RPE cells are lost with age, but this may be compensated for by peripheral cell production, with new cells migrating centrally [4]. This is consistent with results showing that some BrdU-positive cells are located centrally when cell cycle activity is only found in the periphery [3]. This proposition would also be compatible with the results presented here where we show that peripheral RPE cells express an enhanced capacity to divide after injury.

In light of the above, it is reasonable to ask why cell addition in the mature RPE occurs in the periphery rather than at the center where cell death in normal aging has been identified [4]; it would make more biologic sense for cell production to be focused where replenishment is needed. The significance of the periphery in this respect becomes more prominent when developmental and evolutionary aspects of the retina are considered. In vertebrates, all retinal development is initiated centrally and terminates at the periphery [18–21]. Hence, at any developmental stage the central retina is the most mature region and the periphery is the last to adopt senescent qualities. In the case of the teleost eye, the periphery never adopts a senescent state but continues to support a low level of cell production at the retinal margin in both the neural retina and the RPE, giving rise to retinal expansion [1]. This may be the reason why mammals have retained a capacity to produce new RPE and support more efficiently peripheral repair mechanisms. It is also the reason why the interface region between the iris and the retina has been the location from which stem cells have been successfully harvested in mammals [2].

An unexpected finding of this study was the observation that when more than one lesion was made, Ki67 upregulation was found across the entire retina. This was not due to systemic factors as when only one eye was lesioned no upregulation was found in the fellow eye (data not presented). Examining the large number of animal used here with different numbers and patterns of lesioning reveals that there is not a simple relationship between the number of lesions and the extent of this pan retinal upregulation. Irrespective of lesion numbers and patterns only a limited pool of cells entre the cell cycle and divide. This raises the question of whether these cells represent a specific population within the RPE sheet that is ready to proliferate in response to damage, providing the RPE with a trigger mechanism in response to injury independent of location. It also raises the interesting questions: what triggers these cells and by what route of communication do they detect the distant damage?

One potential criticism of our study is that we have only viewed a limited time window following RPE lesions and have not varied lesion size or other parameters. These are valid points; however, the experiments that we undertook addressed the questions that we posed, namely whether there are differences between central and peripheral regions in response to injury. Our time widows were restricted on the basis that we knew from previous pilot studies that they contained the main response to our intervention. Additional data that have been generated regarding widespread RPE proliferation in response to local injury are a bonus, but their exploration raises questions that are separate from those originally addressed here.

While it is clear that lesions repair, they do not do so perfectly. There are two unusual features of the repaired tissue. First, the cells are hypopigmented. Within the repaired region there was variability in levels of pigmentation, but in every case pigmentation was reduced compared to normal cells, and in some cases cells were almost devoid of pigment. This may arise simply because when the cell divides it shares its melanin content between the progeny. If the cell divides several times, the levels of melanin are likely to become very low. However, there is an interesting facet to this argument as during development hypopigmented cells are associated with elevated levels of proliferation compared to those with normal melanin content [22,23]. Hence, while the normal peripheral RPE in DA rats contains approximately 20 cells in the cell cycle, the number in albinos is closer to 200, although the majority of these are polyploid and do not progress to full cell division [3,10] Such differences are likely to be due to the absence of 3,4-dihydroxyphenylalanine (DOPA), which is in the synthetic pathway of melanin and known to slow the pace of the cell cycle and to signal cell-cycle exit [22,23]. The reduction in the level of pigmentation following the first division may increase the probability of subsequent divisions and hence increase the pace of repair. In light of this, it would be interesting to know whether albino RPE repairs at a faster rate than RPE in normally pigmented animals. Unfortunately, such experiments have not been possible because the absorption of the laser that takes place when it hits the pigmented RPE is very different from that in the albino where no melanin is present to absorb the laser energy. Hence, equivalent energy levels have very different impacts on the two tissue types, making direct comparisons impossible. An alternative explanation for the reduced level of pigmentation may come from the finding that transforming growth factor-β, a multifunctional cytokine involved in wound healing, is upregulated following laser lesioning of the RPE [24,25]. Its expression is associated with expression of MMPs, which were shown to be present in lesions induced in this study [26]. Further, transforming growth factor-β is known to decrease melanin synthesis [27].

The second unusual feature of the repaired tissue is that when the neural retina was removed from the RPE, in every case where there had been repair, it was associated with a marked increase of local autofluorescence. Closer examination revealed that this was due to the attachment of sheets of photoreceptor outer segments specific to the repaired region. However, it is known that RPE cells extend long apical processes between outer segments [28] and that these play a role in removing outer segment tips and absorbing them into the cell. The relationship between the RPE and the outer segments can also vary as a function of its adaptive state with light, resulting in a much stronger association between the RPE and the outer segments [28]. Although this does not normally cause problems in attempts to separate these tissues in mammals, such tissue separation can be problematic in some teleosts, amphibians, and birds where dark adaptation is needed. The reason for the tight attachment between the neural retina and the RPE is unclear, but it does indicate an unusual relationship between these repaired tissues.

The RPE is commonly viewed as a relatively homogeneous tissue. A recent report is among the first to suggest that this is not the case [3], and at least on the grounds of proliferative capacity, the RPE can be divided regionally. The data presented in this study confirm our hypothesis that the RPE cell population can be segmented according to its capacity to repair after injury. Current studies in our laboratory are adding significant weight to the notion that this tissue is heterogeneous, with different regions having markedly different levels of expression of markers commonly thought to be in all RPE cells. How RPE cells with different molecular markers respond to challenge or pathological insult will clearly be of importance in understanding this tissue.
